# Association between body mass index and three-year outcome of acute myocardial infarction

**DOI:** 10.1038/s41598-023-43493-0

**Published:** 2024-03-01

**Authors:** Soyoon Park, Dae-Won Kim, Kyusup Lee, Mahn-Won Park, Kiyuk Chang, Myung Ho Jeong, Young Keun Ahn, Sung Chull Chae, Tae Hoon Ahn, Seung Woon Rha, Hyo-Soo Kim, Hyeon Cheol Gwon, In Whan Seong, Kyung Kuk Hwang, Kwon-Bae Kim, Kwang Soo Cha, Seok Kyu Oh, Jei Keon Chae

**Affiliations:** 1 Department of Internal Medicine, Division of Cardiology, Seoul St. Mary’s Hospital, College of Medicine, The Catholic University of Korea, Seoul, Republic of Korea; 2 Department of Internal Medicine, Division of Cardiology, Daejeon St. Mary’s Hospital, College of Medicine, The Catholic University of Korea, Seoul, Republic of Korea; 3 Department of Internal Medicine, Division of Cardiology, Daejeon St. Mary’s Hospital, College of Medicine, The Catholic University of Korea, 222 Banpo daero, Seocho-gu, Seoul, 06591 Republic of Korea; 4 Chonnam National University Hospital, Gwangju, Republic of Korea; 5 Kyungpook National University Hospital, Daegu, Republic of Korea; 6 Gil Medical Center, Gachon University, Incheon, Republic of Korea; 7 Guro Hospital, Korea University, Seoul, Republic of Korea; 8 Seoul National University Hospital, Seoul, Republic of Korea; 9 Samsung Medical Center, Sungkyunkwan Universtiy, Seoul, Republic of Korea; 10 Chungnam National University Hospital, Daejeon, Republic of Korea; 11 Chungbuk National University Hospital, Cheongju, Republic of Korea; 12 Keimyung University Dongsan Medical Center, Daegu, Republic of Korea; 13 Pusan National University Hospital, Busan, Republic of Korea; 14 Wonkwang University Hospital, Iksan, Republic of Korea; 15 Chonbuk National University Hospital, Jeonju, Korea

**Keywords:** Cardiology, Diseases, Health care

## Abstract

Body mass index (BMI), as an important risk factor related to metabolic disease. However, in some studies higher BMI was emphasized as a beneficial factor in the clinical course of patients after acute myocardial infarction (AMI) in a concept known as the “BMI paradox.” The purpose of this study was to investigate how clinical outcomes of patients treated for AMI differed according to BMI levels. A total of 10,566 patients in the Korea Acute Myocardial Infarction Registry-National Institutes of Health (KAMIR-NIH) from May 2010 to June 2015 were divided into three BMI groups (group 1: BMI < 22 kg/m^2^, group 2: ≥ 22 and < 26 kg/m^2^, and group 3: ≥ 26 kg/m^2^). The primary outcome was major adverse cardiac and cerebrovascular event (MACCE) at 3 years of follow-up. At 1 year of follow-up, the incidence of MACCE in group 1 was 10.1% of that in group 3, with a hazard ratio (HR) of 2.27, and 6.5% in group 2, with an HR of 1.415. This tendency continued up to 3 years of follow-up. The study demonstrated that lower incidence of MACCE in the high BMI group of Asians during the 3-year follow-up period compared to the low BMI group. The results implied higher BMI could exert a positive effect on the long-term clinical outcomes of patients with AMI undergoing percutaneous coronary intervention (PCI).

## Introduction

The appropriate control of risk factors affecting the progression of cardiovascular (CV) disease and the incidence of complications is important to improving the clinical outcomes of patients diagnosed with acute myocardial infarction (AMI). Obesity has been considered a risk factor related to ischemic heart disease^1,2^. BMI, a parameter of obesity, has been used to estimate the degree of obesity. According to prior studies, obesity may contribute to atherosclerotic changes by activating inflammatory metabolism^3^. It may also be related to neurohormonal imbalance, predisposing left ventricular remodeling^4^. Higher BMI has been assumed to correlate with higher CV disease occurrence and worse patient prognosis. In contrast, several recent studies showed contrary results on the relationship between BMI and CV disease prognosis, which has been called the “BMI paradox”^5–8^. The relationship was confirmed not only in patients with AMI but also in the general population^9^. Our prior study also found that higher BMI was a protective factor in 1-year all-cause death after AMI^10^. However, large-scale, long-term studies of the BMI paradox concept in Asians are lacking. In this study, we aimed to identify the long-term occurrence of MACCE after AMI during 1-year and 3-year follow-up periods in Asians according to BMI.

## Methods

### Study design and population

**Figure 1:**
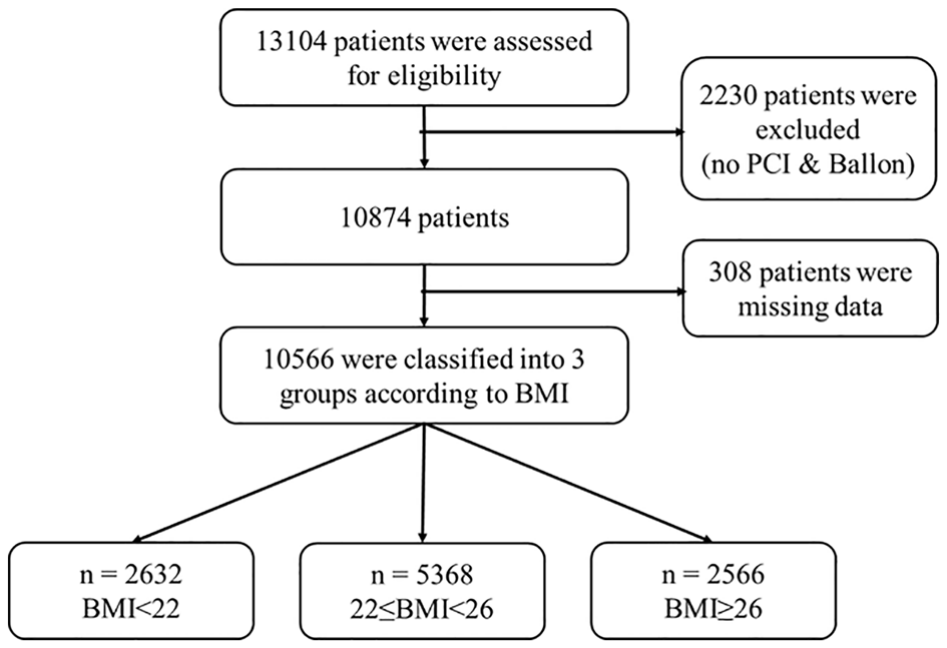
Study population. The Korea Acute Myocardial Infarction Registry-National Institutes of Health (KAMIR-NIH) database. Out of 13,104 patients, 10,566 with AMI who were treated with PCI from May 2010 to June 2015 were enrolled. They were divided into three groups according to BMI. Patients who were not treated with PCI (2230) and 308 patients with missing data were excluded.

The Korea Acute Myocardial Infarction Registry-National Institutes of Health (KAMIR-NIH) database was accessed for this study. Out of 13,104 patients, 10,566 with AMI who were treated with PCI from May 2010 to June 2015 were enrolled. They were divided into three groups according to BMI. Patients who were not treated with PCI (2230) and 308 patients with missing data were excluded (Fig. 1). The KAMIR-NIH was a prospective, multicenter, observational cohort study supported by a grant from 15 Korea Centers for Disease Control and Prevention. All of the centers that participated in the study were high-volume centers familiar with PCI procedures using the standard study protocol. This study was conducted according to the Declaration of Helsinki with the informed consent of the patients and approval of the institutional review board at each participating institution (IRB of the Catholic University of Korea, Daejeon, St. Mary’s hospital, IRB of the Catholic University of Korea, Seoul, St. Mary’s hospital, Gachon Gil Medical Center, IRB of Chonnam National University, Korea University Guro IVD Suppport Center, Seoul National University Hospital Biomedical Research Institute, Samsung Medical Center Clinical Trial Center, IRB of Chungnam National University Hospital, IRB of Chungbuk National University Hospital, IRB of Kyungpook National University Hospital, Clinical Trial Center of Keimyung University Dongsan Medical Center, Clinical Trial Center of Pusan National University Hospital, IRB of Wonkwang University Hospital and IRB of Chonbuk National University Hospital).

### Percutaneous coronary intervention procedure and medical treatment

A standardized procedure protocol based on AMI guidelines was applied to patients diagnosed with AMI. The procedural process and selection of devices among operators were slightly different. However, the same regimen was adopted for pre/postprocedural antiplatelet therapy and periprocedural anticoagulation. Preprocedural antiplatelet therapy included aspirin (200 mg) and clopidogrel (300 or 600 mg), ticagrelor (180 mg), or prasugrel (60 mg). Postprocedural antiplatelet therapy was conducted. Aspirin (100 mg/day) with clopidogrel (75 mg/day), ticagrelor (90 mg twice/day), prasugrel (10 mg/day) was taken for at least 12 months, and after that aspirin (100–200 mg/day) was maintained^11^.

### Definitions and study end-points

We adopted the same definitions described in our prior study. The diagnosis of AMI was based on the value of cardiac biomarkers creatinine kinase-MB and troponin I or T, and other clinical findings, including patient’s symptoms, electrocardiogram (ECG) changes, and imaging, such as transthoracic echocardiogram^12^.

BMI was calculated as weight (kg) divided by height squared (m^2^). As in our previous study, the study groups were equally divided into quartile^10^. BMI was divided into three groups and two additional groups. Group 1 was a BMI of < 22 kg/m^2^, group 2 was 22 to < 26 kg/m^2^, group 3 was ≥ 26 kg/m^2^, with supplementary groups 4 (BMI ≥ 26 to < 30 kg/m^2^) and 5 (≥ 30 kg/m^2^).

The presence of underlying disease was evaluated on admission. Chronic kidney disease (CKD) was defined as a glomerular filtration rate (GFR) upon admission of 60 mL/min per 1.73m^2^, according to the Modification of Diet and Renal Disease Study formula^13^. Fasting glucose concentration of ≥ 7.0 mmol/L, a blood glucose concentration of ≥ 11.0 mmol/L in a 75 g, 2-h oral glucose tolerance test, or the use of antidiabetic therapy was defined as diabetes mellitus (DM). A history of systolic blood pressure ≥ 140 mmHg and a diastolic pressure of ≥ 90 mmHg, or the use of antihypertensive therapy were considered to indicate hypertension (HTN). A fasting total cholesterol concentration of ≥ 220 mg/dL, a fasting triglyceride concentration of ≥ 150 mg/dL, or the use of antihyperlipidemic therapy were regarded as hyperlipidemia.

The primary outcome was MACCE at 3 years of follow-up. The secondary outcome was all-cause death, heart failure, stent thrombosis, target vessel revascularization (TVR), TIMI (thrombolysis in myocardial infarction), and minor bleeding at 3 years of follow-up. MACCE, as the composite of cardiac and cerebrovascular events, included cardiac death, heart disease, and cerebrovascular disease. Death caused by cardiac dysfunction related to ischemic heart disease, heart failure, or arrhythmia, and unwitnessed death was considered to be cardiac death (CD). Death from causes except cardiac disease was defined as all-cause death (AD). MI was defined as mentioned above, and heart failure was considered an ejection fraction of  < 40% during follow-up, with signs and symptoms of heart failure. A cerebrovascular event was defined as a stroke with accompanying neurological impairment lasting longer than 24 h. Percutaneous or surgical revascularization of the stent-inserted lesion, including 5 mm margin segments more proximally or distally, was regarded as TVR. Stent thrombosis was evaluated according to the Academic Research Consortium Definition^14^. Minor bleeding was considered overt clinical bleeding, which was regarded as a fall in hemoglobin less than 3 or equal to 5 g/dL or in hematocrit less than 9% or equal to 15%^15^.

### Statistical analyses

Continuous variables are presented as the mean ± standard deviation and analyzed with the Kruskal–Wallis test. Categorical variables were analyzed by Pearson’s chi-squared test and shown as frequencies and percentages. Analysis of variance (ANOVA) was used to compare BMI groups. Bonferroni’s test was used for post-hoc tests.

The Cox-proportional hazard model was used to evaluate the primary outcome, and the hazard ratio (HR) with a 95% confidence interval (CI) was calculated. All of the variables in Tables 1, 2 and 3 were included and analyzed by univariate analysis. The multivariate logistic model was applied based on the variables in univariate analysis with statistical significance. The primary outcome and secondary outcome incidence in the three groups was compared by the long-rank test and expressed through Kaplan–Meier curves. P-values of < 0.05 were considered statistically significant. All statistical analyses were performed with SAS, version 9.2 (SAS Institute, Cary, NC, USA).

**Table 1:** Baseline demographic, clinical and laboratory characteristics.

Variable	Group 1, N = 2632	Group 2, N = 5368	Group 3, N = 2566	P-value	Group 4, N = 2100	Group 5, N = 466	P-value
Demographics							
Age (years)	69.1 ± 11.7	63.0 ± 11.6	58.1 ± 12.4	< 0.001	58.8 ± 12.1	55.2 ± 13.6	< 0.001
Male	1708 (64.9)	4265 (79.5)	2046 (79.7)	< 0.001	1696 (80.8)	350 (75.1)	0.006
BMI (kg/m^2^)	20.1 ± 1.5	23.9 ± 1.1	28.3 ± 2.3	–	27.5 ± 1.1	32.1 ± 2.5	–
Disease classification							
NSTEMI	1286 (48.9)	2527 (47.1)	1249 (48.7)	0.217	1024 (48.8)	225 (48.3)	0.852
STEMI	1346 (51.1)	2841 (52.9)	1317 (51.3)		1076 (51.2)	241 (51.7)	
Killip							
I	1954 (74.2)	4342 (80.9)	2145 (83.6)	< 0.001	1753 (83.5)	392 (84.1)	0.916
II	262 (10.0)	415 (7.7)	179 (7.0)		150 (7.1)	29 (6.2)	
III	247 (9.4)	324 (6.0)	134 (5.2)		109 (5.2)	25 (5.4)	
IV	169 (6.4)	287 (5.4)	108 (4.2)		88 (4.2)	20 (4.3)	
Risk factors							
Family history of CAD	116 (4.4)	366 (6.8)	215 (8.4)	< 0.001	165 (7.9)	50 (10.7)	0.043
Diabetes mellitus	732 (27.8)	1474 (27.5)	712 (27.8)	0.933	583 (27.8)	129 (27.7)	0.972
Hypertension	1283 (48.8)	2570 (47.9)	1402 (54.6)	< 0.001	1127 (53.7)	275 (59.0)	0.036
Dyslipidemia	195 (7.4)	609 (11.4)	396 (15.4)	< 0.001	325 (15.5)	71 (15.2)	0.897
Current/recent smoker	910 (34.6)	2221 (41.4)	1184 (46.1)	< 0.001	948 (45.1)	236 (50.6)	0.031
Chronic kidney disease	597 (22.7)	905 (16.9)	371 (14.5)	< 0.001	303 (14.4)	68 (14.6)	0.928
Cardiovascular disease history							
Prior myocardial infarction	156 (5.9)	296 (5.5)	162 (6.3)	0.348	127 (6.1)	35 (7.5)	0.240
Prior PCI	211 (8.0)	420 (7.8)	220 (8.6)	0.516	175 (8.3)	45 (9.7)	0.356
Prior CHF	46 (1.8)	42 (0.8)	23 (0.9)	< 0.001	17 (0.8)	6 (1.3)	0.288
Cerebrovascular disease	199 (7.6)	312 (5.8)	128 (5.0)	< 0.001	105 (5.0)	23 (4.9)	0.954
Atrial fibrillation/flutter	143 (5.4)	226 (4.2)	112 (4.4)	0.042	91 (4.3)	21 (4.5)	0.869
Laboratory findings							
HbA1c (%)	6.4 ± 1.6	6.5 ± 1.5	6.6 ± 1.5	< 0.001	6.6 ± 1.5	6.7 ± 1.5	0.247
Pro BNP	3707.0 ± 11,151.6	1725.2 ± 5325.6	1406.4 ± 4299.5	< 0.001	1346.8 ± 4175.5	1673.3 ± 4814.4	0.350
Hb (g/dL)	13.0 ± 2.1	14.1 ± 1.9	14.6 ± 2.0	< 0.001	14.6 ± 1.9	14.7 ± 2.1	0.439
hsCRP (mg/L)	1.7 ± 3.7	1.2 ± 3.1	1.1 ± 2.8	< 0.001	1.1 ± 2.9	0.9 ± 2.2	0.038
Total cholesterol (mg/dL)	172.6 ± 45.4	181.3 ± 44.5	187.5 ± 45.7	< 0.001	186.6 ± 45.7	191.5 ± 45.5	0.007
Triglyceride (mg/dL)	106.7 ± 95.8	135.5 ± 112.6	165.5 ± 124.8	< 0.001	162.3 ± 124.0	179.9 ± 127.7	0.117
LDL cholesterol (mg/dL)	106.6 ± 38.1	114.8 ± 39.2	120.0 ± 39.6	< 0.001	119.4 ± 39.3	122.7 ± 41.0	0.172
HDL cholesterol (mg/dL)	44.9 ± 12.9	42.2 ± 11.1	41.1 ± 10.7	< 0.001	41.2 ± 10.9	40.5 ± 9.4	0.465
In-hospital medications							
Aspirin	2631 (100.0)	5359 (99.8)	2565 (100.0)	0.120	2099 (99.9)	466 (100.0)	> 0.999
Clopidogrel	2159 (82.0)	4095 (76.3)	1904 (74.2)	< 0.001	1556 (74.1)	348 (74.7)	0.795
Ticagrelor or prasugrel	625 (23.8)	1684 (31.4)	897 (35.0)	< 0.001	730 (34.8)	167 (35.8)	0.660
Beta blocker	2105 (80.0)	4574 (85.2)	2276 (88.7)	< 0.001	1862 (88.7)	414 (88.8)	0.914
Calcium channel blocker	139 (5.3)	272 (5.1)	180 (7.0)	0.001	146 (7.0)	34 (7.3)	0.793
ACE inhibitor or ARB	2025 (76.9)	4319 (80.5)	2144 (83.6)	< 0.001	1745 (83.1)	399 (85.6)	0.183
Statin	2386 (90.7)	5029 (93.7)	2434 (94.9)	< 0.001	1996 (95.1)	438 (94.0)	0.350
Oral_anticoagulant (Warfarin)	75 (2.9)	130 (2.4)	59 (2.3)	0.391	51 (2.4)	8 (1.7)	0.354
GP IIb/IIIa inhibitor	324 (12.3)	810 (15.1)	467 (18.2)	< 0.001	372 (17.7)	95 (20.4)	0.176
LVEF	50.2 ± 11.4	52.2 ± 10.5	53.5 ± 10.3	0.001	53.6 ± 10.2	53.2 ± 10.8	0.203
LVEF < 40%	419 (15.9)	568 (10.6)	216 (8.4)	< 0.001	175 (8.3)	41 (8.8)	0.744

Data are presented as mean ± SD, number (percentage). BMI was divided to BMI range (Group 1 ≤ 22 kg/m^2^, Group 2 ≥ 22 < 26 kg/m^2^ and Group 3 ≥ 26 kg/m^2^–Group 4 ≥ 26 < 30 kg/m^2^ Group 5 ≥ 30 kg/m^2^). P-value less than 0.005 is statistically significant.

*BMI* body mass index, *STEMI* ST segment elevation myocardial infarction, *NSTEMI* non-ST segment elevation myocardial infarction, *CAD* coronary artery disease, *CKD* chronic kidney disease, *CHF* congestive heart failure, *PCI* percutaneous coronary intervention, *Hb* hemoglobin, *hsCRP* high sensitivity C-reactive protein, *LDL cholesterol* low density lipoprotein cholesterol, *HDL cholesterol* high density lipoprotein Cholesterol, *ACE inhibitor* angiotensin-converting enzyme inhibitor, *ARB* angiotensin receptor blocker, *Gp IIb*/*IIIa* inhibitor glycoprotein IIb/IIIa inhibitor, *LVEF* left ventricular ejection fraction.

**Table 2:** The characteristics of procedure.

Variable	Group 1, N = 2632	Group 2, N = 5368	Group 3, N = 2566	P-value	Group 4, N = 2100	Group 5, N = 466	P-value
Target vessel (%)							
LAD	1276 (48.5)	2549 (47.5)	1163 (45.3)	0.252	956 (45.5)	207 (44.4)	0.917
LCX	426 (16.2)	908 (16.9)	455 (17.7)		372 (17.7)	83 (17.8)	
RCA	866 (32.9)	1790 (33.4)	897 (35.0)		729 (34.7)	168 (36.1)	
LM	64 (2.4)	121 (2.3)	51 (2.0)		43 (2.1)	8 (1.7)	
Number of diseased vessels (%)							
1	1239 (47.1)	2618 (48.8)	1293 (50.4)	0.084	1044 (49.7)	249 (53.4)	0.252
2	774 (29.4)	1596 (29.7)	757 (29.5)		619 (29.5)	138 (29.6)	
3	493 (18.7)	896 (16.7)	403 (15.7)		343 (16.3)	60 (12.9)	
Lesion classification (%)							
A	31 (1.2)	70 (1.3)	33 (1.3)	0.666	27 (1.3)	6 (1.3)	0.937
B1	321 (12.2)	646 (12.0)	284 (11.1)		236 (11.2)	48 (10.3)	
B2	1001 (38.0)	1974 (36.8)	985 (38.4)		807 (38.4)	178 (38.2)	
C	1279 (48.6)	2678 (49.9)	1264 (49.3)		1030 (49.1)	234 (50.2)	
Pre-PCI TIMI 0 or 1, n (%)	1441 (54.8)	3061 (57.0)	1516 (59.1)	0.007	1235 (58.8)	281 (60.3)	0.554
Post-PCI TIMI 0 or 1, n (%)	10 (0.4)	14 (0.3)	7 (0.3)	0.636	4 (0.2)	3 (0.6)	0.118
Post-PCI TIMI 3, n (%)	2549 (96.9)	5218 (97.2)	2507 (97.7)	0.168	2054 (97.8)	453 (97.2)	0.435
Total number of stents (%)	1.5 ± 0.8	1.5 ± 0.8	1.5 ± 0.8	0.649	1.5 ± 0.8	1.4 ± 0.7	0.277
Total stent length	29.9 ± 14.2	29.5 ± 14.3	29.1 ± 13.9	0.189	29.0 ± 13.9	29.8 ± 14.3	0.290
Mean stent diameter	3.0 ± 0.5	3.0 ± 0.5	3.1 ± 0.6	< 0.001	3.1 ± 0.6	3.2 ± 0.6	0.011

Data are presented as mean ± SD, number (percentage). BMI was divided to BMI range (Group 1 ≤ 22 kg/m^2^, Group 2 ≥ 22 < 26 kg/m^2^ and Group 3 ≥ 26 kg/m^2^–Group 4 ≥ 26 < 30 kg/m^2^ Group 5 ≥ 30 kg/m^2^). P-value less than 0.005 is statistically significant.

Lesion based on American College of Cardiology/American Heart Association lesion classification. *LAD* left anterior descending artery, *LCX* left circumflex artery, *LMCA* left main coronary artery, *TIMI* thrombolysis in myocardial infarction.

**Table 3:** Primary and secondary clinical outcomes in AMI patients stratified by BMI at 1-year and 3-year.

	Group		P-value	Log rank P-value	HR (95% CI)	p-value	Adjusted HR (95%CI)	P-value
1-year								
MACCE	Group 1	266 (10.1)	< 0.001	< 0.001	2.270 (1.828–2.818)	< 0.001	1.274 (1.014–1.601)	0.038
	Group 2	348 (6.5)			1.415 (1.149–1.742)	0.001	1.210 (0.980–1.495)	0.077
	Group 3	119 (4.6)			1.000		1.000	
CD	Group 1	183 (7.0)	< 0.001	< 0.001	3.345 (2.475–4.522)	< 0.001	1.518 (1.107–2.081)	0.010
	Group 2	191 (3.6)			1.673 (1.239–2.258)	0.001	1.325 (0.976–1.799)	0.072
	Group 3	55 (2.1)			1.000		1.000	
MI	Group 1	50 (1.9)	0.143	0.091	1.597 (1.024–2.488)	0.039	1.090 (0.677–1.756)	0.723
	Group 2	79 (1.5)			1.195 (0.792–1.801)	0.396	1.082 (0.712–1.643)	0.713
	Group 3	32 (1.3)			1.000		1.000	
TVR	Group 1	13 (0.5)	0.334	0.417	0.636 (0.319–1.279)	0.200	0.807 (0.381–1.709)	0.575
	Group 2	39 (0.7)			0.899 (0.529–1.529)	0.695	1.008 (0.583–1.745)	0.977
	Group 3	21 (0.8)			1.000		1.000	
CVA	Group 1	35 (1.3)	0.053	0.037	2.107 (1.180–3.761)	0.012	1.332 (0.719–2.467)	0.362
	Group 2	60 (1.1)			1.712 (0.999–2.933)	0.050	1.468 (0.849–2.539)	0.169
	Group 3	17 (0.7)			1.000		1.000	
AD	Group 1	263 (10.0)	< 0.001	< 0.001	3.413 (2.651–4.394)	< 0.001	1.543 (1.184–2.011)	0.001
	Group 2	267 (5.0)			1.651 (1.283–2.124)	< 0.001	1.327 (1.026–1.717)	0.031
	Group 3	78 (3.0)			1.000		1.000	
NEW-HF	Group 1	150 (5.7)	< 0.001	< 0.001	1.967 (1.491–2.595)	< 0.001	1.073 (0.799–1.442)	0.638
	Group 2	202 (3.8)			1.290 (0.990–1.682)	0.059	1.109 (0.846–1.452)	0.454
	Group 3	75 (2.9)			1.000		1.000	
ST	Group 1	12 (0.5)	0.082	0.070	4.066 (1.148–14.405)	0.030	4.177 (1.095–15.940)	0.036
	Group 2	19 (0.4)			3.061 (0.906–10.340)	0.072	3.263 (0.945–11.267)	0.062
	Group 3	3 (0.1)			1.000		1.000	
Minor bleeding	Group 1	111 (4.2)	< 0.001	< 0.001	1.992 (1.442–2.752)	< 0.001	1.784 (1.259–2.528)	0.001
	Group 2	145 (2.7)			1.264 (0.927–1.724)	0.139	1.223 (0.891–1.677)	0.213
	Group 3	55 (2.1)			1.000		1.000	
3-year								
MACCE	Group 1	412 (15.7)	< 0.001	< 0.001	2.097 (1.774–2.478)	< 0.001	1.230 (1.030–1.469)	0.022
	Group 2	600 (11.2)			1.416 (1.209–1.658)	< 0.001	1.217 (1.037–1.429)	0.016
	Group 3	207 (8.1)			1.000		1.000	
CD	Group 1	271 (10.3)	< 0.001	< 0.001	3.643 (2.828–4.692)	< 0.001	1.583 (1.215–2.062)	0.001
	Group 2	274 (5.1)			1.725 (1.340–2.221)	< 0.001	1.332 (1.030–1.721)	0.029
	Group 3	77 (3.0)			1.000		1.000	
MI	Group 1	90 (3.4)	0.196	0.074	1.446 (1.052–1.987)	0.023	1.109 (0.787–1.563)	0.556
	Group 2	167 (3.1)			1.234 (0.928–1.641)	0.148	1.141 (0.853–1.527)	0.374
	Group 3	66 (2.6)			1.000		1.000	
TVR	Group 1	27 (1.0)	0.066	0.139	0.682 (0.421–1.106)	0.121	0.892 (0.529–1.504)	0.668
	Group 2	90 (1.7)			1.045 (0.725–1.508)	0.813	1.182 (0.809–1.726)	0.387
	Group 3	42 (1.6)			1.000		1.000	
CVA	Group 1	63 (2.4)	0.146	0.061	1.596 (1.080–2.359)	0.019	1.027 (0.676–1.560)	0.902
	Group 2	115 (2.1)			1.339 (0.940–1.906)	0.106	1.182 (0.824–1.695)	0.363
	Group 3	42 (1.6)			1.000		1.000	
AD	Group 1	417 (15.8)	< 0.001	< 0.001	3.638 (2.970–4.457)	< 0.001	1.576 (1.274–1.949)	< .0001
	Group 2	430 (8.0)			1.742 (1.423–2.133)	< 0.001	1.348 (1.097–1.655)	0.004
	Group 3	120 (4.7)			1.000		1.000	
NEW-HF	Group 1	150 (5.7)	< 0.001	< 0.001	1.967 (1.491–2.595)	< 0.001	1.073 (0.799–1.442)	0.638
	Group 2	202 (3.8)			1.290 (0.990–1.682)	0.059	1.109 (0.846–1.452)	0.454
	Group 3	75 (2.9)			1.000		1.000	
ST	Group 1	19 (0.7)	0.725	0.591	1.433 (0.718–2.858)	0.307	1.430 (0.671–3.047)	0.354
	Group 2	35 (0.7)			1.218 (0.655–2.264)	0.532	1.296 (0.683–2.458)	0.428
	Group 3	14 (0.6)			1.000		1.000	
Minor bleeding	Group 1	112 (4.3)	< 0.001	< 0.001	2.010 (1.456–2.776)	< 0.001	1.804 (1.274–2.555)	0.001
	Group 2	145 (2.7)			1.264 (0.927–1.724)	0.139	1.224 (0.892–1.679)	0.211
	Group 3	55 (2.1)			1.000		1.000	

Data are presented as n (%), *CI* confidence interval; *HR* hazard ratio. All of the variables in Table 3 were included and analyzed to perform univariate analysis. On the basis of the variables that were significant (*P* < 0.05) according to univariate analysis, a multivariate Cox proportional hazard model was constructed. *CD* cardiac death, *AD* all cause death except cardiac death, *MI* myocardial infarction, *CVA* cerebrovascular attack, *TVR* target vessel revascularization, *ST* stent thrombosis.

## Results

### Baseline characteristics of the study population

The low BMI group in the present study included old-aged patients with underlying diseases, such as CKD, prior history of congestive heart failure (CHF), cerebrovascular disease, and atrial fibrillation or flutter. The high BMI group had more CV risk factors, such as dyslipidemia, family history of coronary artery disease (CAD), HTN, and current smoking. There were no statistical differences among the groups in the presence of DM and past PCI treatment or prior MI history among the groups (Table 1). The location and number of lesions and TIMI grade, were not significantly different (Table 2).

### Clinical outcomes of the study population

**Figure 2:**
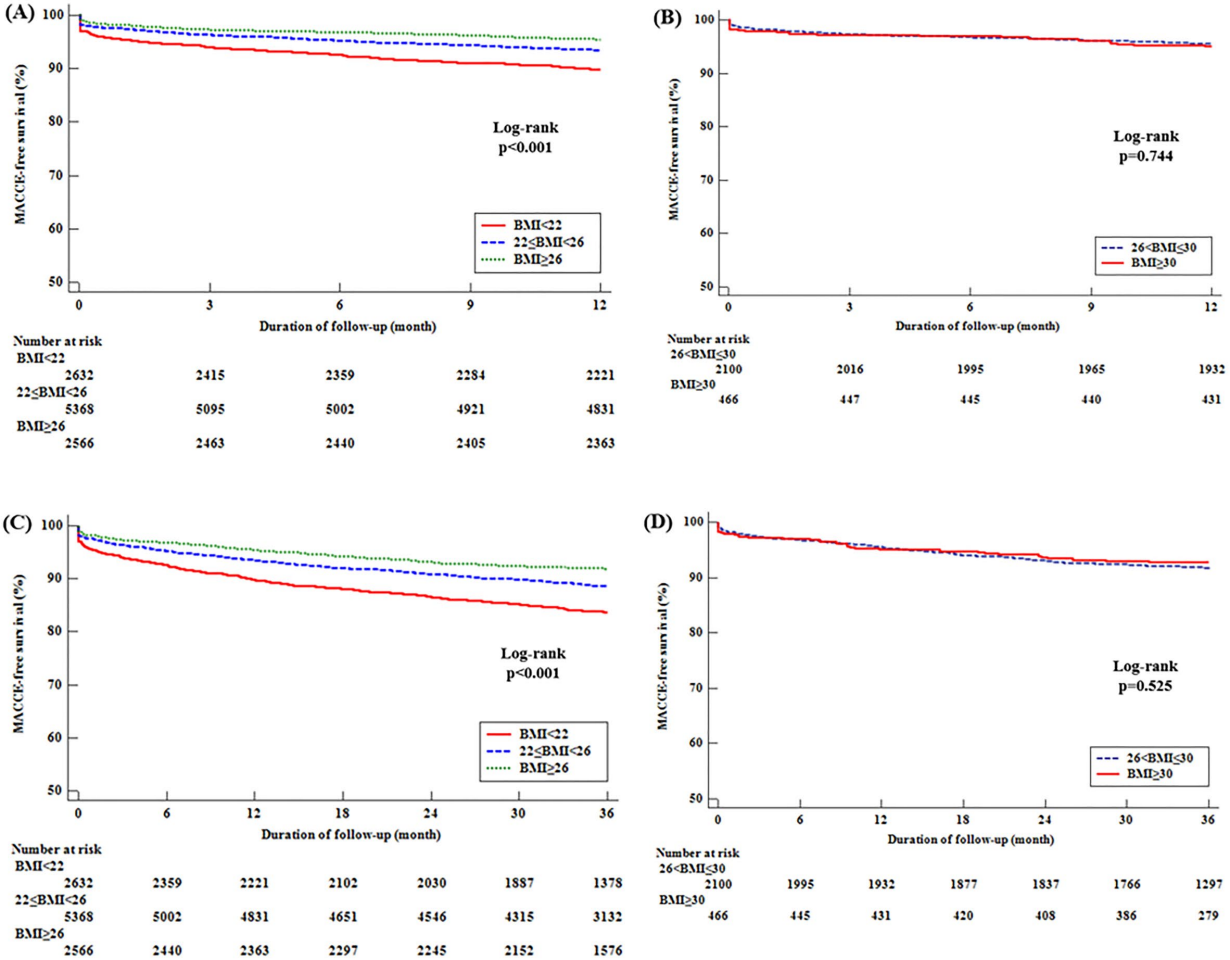
The probability of MACCE-free survival on Kaplan–Meier curves. At 1 year and 3 year follow-up period, the low BMI group was more susceptible to MACCE than the high BMI group (long rank, P < 0.001) (**A**,**C**). However, the probability between groups 4 and 5 was not significantly different (long rank P = 0.744, long rank, P = 0.525, respectively) (**B**,**D**).

At 1 year of follow-up, the incidence of MACCE were higher in low BMI group than high BMI group comparing to group 3 (group 1; 266 [10.1%] vs group 2; 348 [6.5%], P < 0.001) and the results were continued up to 3 years of follow-up (MACCE;aHR 1.230, [1.030–1.469], P = 0.022, CD;aHR 1.583, [1.215–2.062]). In the lower BMI group, the greater increase in MACCE and CD incidence at 3 years of follow-up was identified, which showed incremental effects on MACCE and CD with time according to the BMI. At 1 year of follow-up, in multivariate regression analysis, low BMI behaved as a risk factor related to the incidence of MACCE, CD, AD, and minor bleeding (MACCE; group 1 aHR = 1.274 [1.014–1.601], P = 0.038; Table 3). CD and AD played a major role in the meaningful results among the groups (aHR = 1.518 [1.107–2.081], P = 0.010, aHR = 1.543, [1.184–2.011], P = 0.001; Table 3). Other components, such as the incidence of MI, TVR, CVA, and new-onset HF and ST, were not statistically significantly different among the three groups. The incidence of ST and minor bleeding events was higher in the lower BMI group (ST: aHR = 4.177 [1.095–15.940], P = 0.036; minor bleeding: aHR = 1.784, [1.259–2.528, P = 0.001], but the difference was not statistically significant at 3 years of follow-up (Table 3). The probability of MACCE-free survival was shown on Kaplan–Meier curves. The low BMI group was more susceptible to MACCE than the high BMI group.However, the probability between groups 4 and 5 was not significantly different (Fig. 2A–D).

### Predictors of overall mortality

Univariable and multivariable logistic regression analyses were conducted to evaluate the independent predictive factors influencing the primary outcome. Killip classification, DM, HTN, smoking history, CKD, cerebrovascular disease, atrial fibrillation, the use of beta blockers, ACE or ARB inhibitors, and statins, age, Hb, hsCRP, LVED, and stent diameter were identified as the independent predictors of the primary outcome (Table 4).

**Table 4:** Multivariate analysis for overall mortality.

NSTEMI	Adjusted OR	95% CI		P-value		
		Lower	Upper			
Age	1.025	1.018	1.032	< 0.001		
CKD	1.287	1.092	1.517	0.003		
DM	1.205	1.034	1.404	0.017		
HTN	1.307	1.134	1.505	< 0.001		
Cerebrovascular disease	1.303	1.042	1.629	0.020		
Atiral fibrillation	1.337	1.031	1.733	0.028		
Smoking	1.176	1.005	1.375	0.043		
Use of beta blocker	0.655	0.554	0.774	< 0.001		
Use of ACE inhibitor/ARB	0.715	0.611	0.838	< 0.001		
Use of Statin	0.348	0.286	0.424	< 0.001		
Hb	0.941	0.906	0.977	0.002		
proBNP	1.000	1.000	1.000	0.005		
hsCRP	1.023	1.004	1.042	0.019		
LVEF	0.990	0.984	0.996	0.001		
Killip classification						
Killip 2 vs 1	1.186	0.954	1.475	0.124	0.0011	
Killip 3 vs 1	1.280	1.025	1.599	0.029		
Killip 4 vs 1	1.562	1.227	1.989	< 0.001		
Target vessel						
LCx vs LAD	0.769	0.632	0.937	0.009	< 0.001	
RCA vs LAD	0.931	0.800	1.084	0.357		
LM vs LAD	2.757	1.927	3.945	< 0.001		
Number of vessels	1.126	1.039	1.222	0.004		
Mean stent diameter	0.754	0.658	0.865	< 0.001		

*OR* odds ratio, *CI* confidence interval, *CKD* chronic kidney disease, *ACE inhibitor* angiotensin-convertingenzyme inhibitor, *ARB* angiotensin receptor blocker, *Hb*  hemoglobin, *HDL cholesterol* high density lipoprotein cholesterol, *TIMI* thrombolysis in myocardial infarction, *LVEF* left ventricular ejection fraction.

Multivariable logistic regression analyses were carried out to identify independent predictors for overall mortality and on the basis of the variables that were significant (P < 0.05) according to univariable logistic regression analysis.

### Subgroup analysis

**Figure 3:**
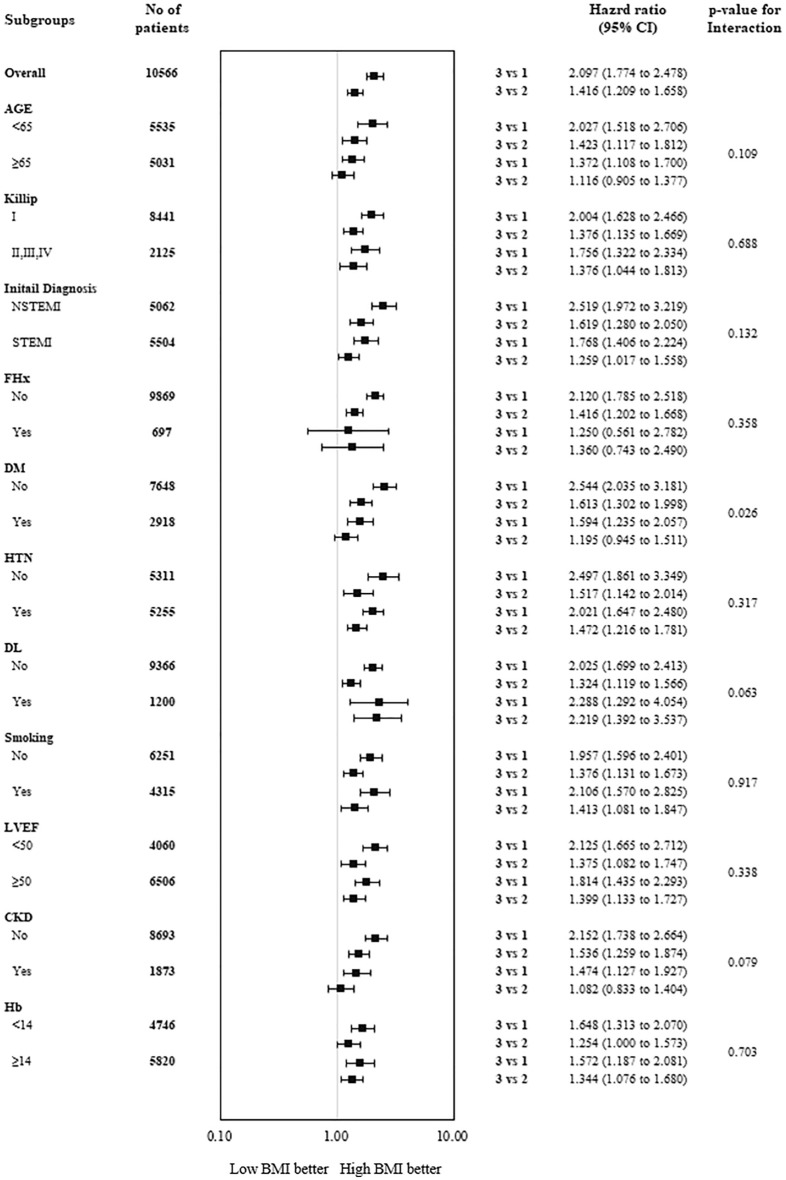
Subgroup analysis of the primary outcome. Hazard ratios and 95% confidence intervals are shown for the primary composite end point of cardiac death, myocardial infarction, target vessel revascularization and cerebrovascular events in subgroups of patients assigned to BMI group. The P- value for interaction represents the likelihood of interaction between the variable and the relative BMI effect.

The positive effect of high BMI on the primary outcome was maintained in subgroup analysis regardless of the independent predictive factors, except DM. When stratified by DM, patients with low BMI without DM had statistically higher risks of MACCE incidence (HR = 2.544 [2.035–3.181], P = 0.026) than those with DM (HR = 1.594, [1.235–2.057], P = 0.026). A marginal interaction with dyslipidemia was seen (P = 0.063 for interaction) (Fig. 3).

## Discussion

Our previous study showed lower all-cause death and cardiac death high BMI group during 1 year of follow-up^10^. Analyzing MACCE as the primary outcome and additional meaningful implications were possible using the detailed information included in the KAMIR registry. First, group 1 showed a higher incidence of MACCE than group 3 at 1 year of follow-up, and it was maintained at 3 years of follow-up. Group 2 also showed poor prognosis in the primary outcome at 3 years of follow-up, although the difference was not statistically significant at 1 year of follow-up. The better clinical outcome in the higher BMI groups and the discrepancy of the results according to the follow-up period in group 2 might be attributed to differences in the incidence of CD. (Table 3) The tendency toward increases in the absolute number of MACCE events over time also showed a positive relationship with CD occurrence. High BMI could be considered a protective factor in the occurrence of CD and MACCE because the results remained after adjustment for other confounding factors. Second, higher BMI had a positive effect on the incidence of AD with statistical significance (417 in group 1, 430 in group 2, and 120 in group 3) than that of our previous study (262 in group 1, 261 in group 2, 77 in group 3)^10^. Third, at 1 year of follow-up, the incidence of ST was significantly higher in group 1 than in group 3, but not at 3 years of follow-up. A prior study identified several risk factors related to the incidence of ST^16^. Statistically significant differences in the baseline characteristics among the groups, such as CKD and heart failure, might have affected ST incidence even after further adjustment. Also, in older people, especially those ≥ 65 years of age, medication compliance could be decreased due to concerns related to adverse reactions with antiplatelet agents^17^. Compliance with medication-taking might have been poor in group 1 because it was the oldest age (group 1, 69.1 ± 11.7 years; group 2, 63.0 ± 11.6 years; and group 3, 58.1 ± 12.4 years, P < 0.001). Clopidogrel was prescribed more often than ticagrelor or prasugrel in group 1 and vice versa in the case of groups 2 and 3. Different drug potencies could be one reason for the differences during 1 year of follow-up because taking DAPT for 1 year after PCI is generally recommended^11^. The incidence of new-onset HF was higher in group 1 than in group 3, and the proportion of prescription medications was higher in group 3 than in group 1. Therefore, it is necessary to closely monitor cardiac function and prescribe appropriate medications to improve cardiac function and long-term prognosis. The incidence of MI, TVR, and CVA was not significantly different among the BMI groups, perhaps due to the small number of cases. Finally, more minor bleeding events occurred in group 1 than in group 3 at both 1 and 3 years of follow-up. The characteristics of the patients in group 1, including low body weight, old age (≥ 65 years), and underlying disease, could predispose them to bleeding events (Table 1).

In subgroup analysis, poor clinical outcomes were identified in the low BMI groups. In particular, low BMI had worse effects on the clinical outcomes in patients without DM than with DM (Fig. 3). It is possible that the accumulation of central fat in DM patients offsets the positive effects of a high BMI^18,19^. Also, considering that the HbA1C levels were lowest in group 1, there might be few DM patients with low BMIs, and the different number of DM patients between the low and high BMI groups might have influenced the HRs. In addition, the primary outcomes in groups 4 and 5 were not statistically significantly different because the number of patients classified into these groups was insufficient to demonstrate statistically significant results.

The positive effect on clinical outcomes in the high BMI groups could be explained by several theories. First, it is possible that patients classified into the low BMI groups had unhealthy metabolic status with cachexic status. Second, as mentioned above, patients in the high BMI group had a tendency to be actively prescribed medication. The regular prescription of medications and appropriate post-PCI monitoring might have had positive effects on their long-term clinical prognosis.

The study had several limitations. First, it inevitably had the limitations of a nonrandomized retrospective study. Second, it is questionable whether BMI can adequately reflect metabolic status. In previous studies, obesity was divided into metabolically “healthy” and “unhealthy” groups. Total body fat accumulation, especially abdominal fat related to metabolic syndrome, was an important factor in the clinical prognosis^20^. Because the registry of the study did not include information on peripheral fat deposition, it could act as a confounding factor. However, despite the limitation, the effect of BMI on the primary outcome could have clinical implications, as several other studies reported a positive correlation between BMI and abdominal circumference^21,22^. An additional limitation is that the proportion of Asian patients with extreme obesity was too small to conclude statistically significant outcomes. Based on the Korea-NIH data, only 0.89% of the general population was classified as having class III obesity^23^. This is why large-scale studies, including other Asian countries besides Korea, are needed in the future. In spite of these limitations, the study has clinically significant implications. First, it was a large-scale study in Asians and it showed 3-year long-term clinical outcomes. Second, the study demonstrated meaningful results in that the study assessed clinical outcomes, which included not only all-cause mortality and cardiovascular events but also cerebrovascular events and various clinical events such as minor bleeding and stent thrombosis. Additionally, we demonstrated independent predictors of overall mortality and evaluated whether the effect of BMI on clinical outcomes was influenced by the independent predictors.

## Conclusion

The present large-scale study showed a lower incidence of MACCE in the high BMI group of Asians during the 3-year follow-up period compared to the low BMI group. In conclusion, a high BMI had a protective effect on long-term clinical outcomes in patients with AMI undergoing PCI, and strict monitoring might be essential for low BMI groups.

## Data Availability

The present study analyzed the KAMIR-NIH data in South Korea. The data are accessible to any researchers after permission of the Disease Control and Prevention and the Korea Health Technology R & D Project, Ministry of Health & Welfare (NIH URL http://icreat.nih.go.kr).
